# Carfilzomib activates ER stress and JNK/p38 MAPK signaling to promote apoptosis in hepatocellular carcinoma cells: Carfilzomib promotes HCC cell death

**DOI:** 10.3724/abbs.2024040

**Published:** 2024-04-09

**Authors:** Yao Deng, Yujie Li, Mingyue Yang, Yang Gao, Xuling Luo, Hanbin Chen, Meng Guo, Xuefeng Yang, Yongzhang Liu, Jun He, Bin Lu, Naxin Liu

**Affiliations:** 1 Department of Gastroenterology and Hunan Provincial Clinical Research Center for Metabolic Associated Fatty Liver Disease The Affiliated Nanhua Hospital and Department of Cell Biology and Genetics School of Basic Medical Sciences Hengyang Medical School University of South China Hengyang 421001 China; 2 School of Laboratory Medicine and Life Sciences Wenzhou Medical University Wenzhou 325035 China; 3 Department of Gastrointestinal Surgery The First Affiliated Hospital Wenzhou Medical University Wenzhou 325000 China; 4 School of Public Health Fudan University Shanghai 200032 China; 5 Department of Oncology The First Affiliated Hospital Wenzhou Medical University Wenzhou 325000 China

**Keywords:** hepatocellular carcinoma, carfilzomib, ER stress, JNK/p38 MAPK signaling, proteasome inhibitor

## Abstract

Hepatocellular carcinoma (HCC) is one of the most prevalent and deadly cancers in the world, which is frequently diagnosed at a late stage. HCC patients have a poor prognosis due to the lack of an efficacious therapeutic strategy. Approved drug repurposing is a way for accelerating drug discovery and can significantly reduce the cost of drug development. Carfilzomib (CFZ) is a second-generation proteasome inhibitor, which is highly efficacious against multiple myeloma and has been reported to possess potential antitumor activities against multiple cancers. However, the underlying mechanism of CFZ on HCC is still unclear. Here, we show that CFZ inhibits the proliferation of HCC cells through cell cycle arrest at the G2/M phase and suppresses the migration and invasion of HCC cells by inhibiting epithelial-mesenchymal transition. We also find that CFZ promotes reactive oxygen species production to induce endoplasmic reticulum (ER) stress and activate JNK/p38 MAPK signaling in HCC cells, thus inducing cell death in HCC cells. Moreover, CFZ significantly inhibits HCC cell growth in a xenograft mouse model. Collectively, our study elucidates that CFZ impairs mitochondrial function and activates ER stress and JNK/p38 MAPK signaling, thus inhibiting HCC cell and tumor growth. This indicates that CFZ has the potential as a therapeutic drug for HCC.

## Introduction

Hepatocellular carcinoma (HCC) occurs predominantly in patients with chronic liver disease and cirrhosis [ 1– 3]. Early clinical features of HCC patients are mainly inconspicuous, and patients are often diagnosed at an advanced stage. Currently, serious adverse reactions frequently occur in advanced HCC patients after treatment with the first-line drugs, such as sorafenib and lenvatinib [4]. Therefore, it is urgently needed to develop safe and more efficacious drugs against HCC.

Carfilzomib (CFZ) is a second-generation selective proteasome inhibitor which was approved for the treatment of relapsed or refractory multiple myeloma by the FDA in 2012 and has a significant safety advantage over bortezomib [5]. In addition to hematological malignancies, CFZ also suppresses the growth of solid tumors, such as neuroblastoma, small cell lung cancer, and anaplastic thyroid cancer (ATC) [ 6– 8]. Lee *et al*. [6] reported that CFZ inhibits the viability of human neuroblastoma cells by promoting apoptosis, which is probably associated with endoplasmic reticulum (ER) stress, reactive oxygen species (ROS) production, and mitochondrial membrane potential changes. Additionally, CFZ was found to inhibit the production of serum tumor markers in diethylnitrosamine-induced hepatocarcinogenesis [9]. CFZ inhibits ATC cell proliferation and reduces the metastasis and progression of ATC *in vivo* [7]. Recently, it was reported that the combination of CFZ and sorafenib has synergistic activities in suppressing cell proliferation, migration, and invasion through inducing apoptosis and inhibiting epithelial-mesenchymal transition (EMT) in HCC cells [10]. Although CFZ has been used in cell and animal studies for the treatment of solid tumors, the underlying mechanisms remain poorly understood, which strongly limits the clinical application of CFZ as an anticancer drug. The proteasome is responsible for degrading 80% of proteins in eukaryotic cells [11]. CFZ irreversibly binds to and inhibits the chymotrypsin-like activity of the 20S catalytic core subunit of the proteasome [12]. Proteasome inhibition can induce cell death by impairing protein degradation and promoting abnormal (damaged and unfolded/misfolded) protein accumulation. The accumulation of abnormal proteins triggers the unfolded protein response (UPR) in the ER, termed UPR ^ER^ [13]. The mammalian UPR ^ER^ is initiated by the activation of three ER transmembrane proteins, including transcription factor (ATF6), inositol requiring kinase 1 (IRE1α), and PKR-like ER kinase (PERK) [14]. IRE1α has both Ser/Thr protein kinase and endoribonuclease activities which are important to the enforcement of UPR and contribute to activation of the c-Jun N-terminal protein kinase (JNK) [ 15, 16]. However, whether CFZ suppresses HCC cell proliferation, migration and invasion via enhancing IRE1α-associated UPR ^ER^ and JNK/p38 MAPK signaling remains unresolved.

In this study, we reveal that CFZ activates ER stress and JNK/p38 MAPK signaling to suppress cell proliferation, migration, and invasion while inducing apoptosis of HCC cells, which suggests that CFZ may be a potential therapeutic agent for treating and improving the outcome of patients with HCC.

## Materials and Methods

### Cell lines and cell culture

HCCLM3, Huh7 and MHCC97H cell lines were purchased from the Chinese Academy of Science Cell Bank (Shanghai, China) and cultured in DMEM (Thermo Fisher Scientific, Waltham, USA) supplemented with 10% FBS (ExCell Bio, Shanghai, China), at 37°C in a humidified incubator with 5% CO _2_. All HCC cell lines were routinely tested and confirmed to be mycoplasma-free in this work.

### Reagents and antibodies

CFZ was purchased from Aladdin Biochemical Technology (Shanghai, China) and dissolved in DMSO prior to use. Annexin V-fluorescein isothiocyanate (FITC)/propidium iodide (PI) Apoptosis Double Dye Detection Kit and PI/RNase Staining Solution were obtained from BD Biosciences (San Jose, USA). Protease inhibitor cocktail was from APExBIO (Houston, USA). Cell Counting Kit-8 (CCK-8), Crystal Violet Staining Solution, and N-acetyl cysteine (NAC) were purchased from Beyotime Biotechnology (Shanghai, China). Pierce BCA ^TM^ Protein Assay Kit, Pierce ^TM^ ECL Western, and TRIzol reagent were purchased from Thermo Fisher Scientific (Waltham, USA). Mayer’s hematoxylin solution was purchased from Solarbio (Beijing, China). Anti-β-actin antibody (P30002) was purchased from Abmart (Shanghai, China). Anti-Ki67 (ab16667), anti-PARP (ab191217), and anti-XBP1 (ab198999) antibodies were purchased from Abcam (Cambridge, UK). Anti-Retinoblastoma (Rb) (A3618) and anti-p-CDK4 (AP0593) antibodies were purchased from Abclonal (Wuhan, China). Anti-CDK1 and anti-CDK4 antibodies were purchased from Bio-Rad (Hercules, USA). Anti-ATF4 (11815), anti-BiP (3117), anti-CHOP (5554), anti-COX IV (4850), anti-Caspase3 (14220), anti-cleaved-Caspase3 (9664), anti-IRE1α (3294), anti-JNK (9252), anti-N-Cadherin (13116), anti-p21 (2947), anti-p38 (8690), anti-p-p38 (4511), anti-p-Rb (8516), and anti-Snail (3879) antibodies were purchased from Cell Signaling Technology (Beverly, USA). Anti-COX II (55070-1-AP), anti-PCNA (10205-2-AP), and anti-SDHA (14865-1-AP) antibodies were purchased from ProteinTech (Wuhan, China).

### Measurement of cell viability and proliferation

Cell viability and proliferation were determined by CCK-8 assay as described previously [17]. Briefly, HCC cells (5×10 ^3^ cells/well) were seeded into a 96-well plate and incubated overnight. Cells were then treated with DMSO or CFZ (5, 10, 20, 40, 80, or 160 nM). After 48 h, the cell viability was determined using CCK-8 according to the manufacturer’s protocol. For the cell proliferation assay, HCC cells (3×10 ^3^ cells/well) were seeded into a 96-well plate and incubated overnight. The next day, cells were treated with DMSO or CFZ (30, 60, or 120 nM) for 1, 2, 3, or 4 days, and proliferation rates were also measured by CCK-8 assay.

### Colony formation assay

Cells were plated into 6-well plates at a density of 1000 cells per well and incubated at 37°C with 5% CO _2_ overnight. The cells were then incubated in DMEM containing vehicle (DMSO) or CFZ (30, 60, or 120 nM) until colonies were visible. During this period, DMEM containing vehicle or CFZ was changed every two days. The cell colonies were stained with 0.5% crystal violet (Beyotime Biotechnology) for 20 min at room temperature, and the number of colonies was counted using ImageJ Plus after images were captured with a digital camera (DSC-WX700; Sony, Tokyo, Japan).

### Transwell migration and invasion assays

Transwell migration and invasion assays were conducted as described previously with slight modification [18]. To perform the *in vitro* cell migration assay, HCCLM3 and MHCC97H cells (4×10 ^4^) in DMEM without FBS were plated in the upper chamber of the transwell plate (Corning Inc., Kennebunk, USA) and 600 μL of DMEM with 10% FBS was added in the lower chamber. The plate was incubated in a humidified cell culture incubator at 37°C with 5% CO _2_ overnight, and then the medium in the upper chamber was replaced by FBS-free medium containing vehicle (DMSO) or CFZ (30, 60, or 120 nM). The cells were further incubated for 48 h, and the migrated cells were stained with 0.5% crystal violet solution. Images of five fields in each well were randomly selected and captured with an inverted microscope. The number of migrated cells was quantified by ImageJ Plus. *In vitro* cell invasion assays were performed using transwell invasion chambers (Corning Inc.) coated with Matrigel (40 μL per filter; 356234; BD Biosciences) as described in the manufacturer’s instructions. The remaining steps were the same as those for the cell migration assay.

### Cell cycle distribution and apoptosis assays

Cells were seeded in 60-mm cell culture dishes and cultured at 37°C and 5% CO _2_ overnight. The next day, the cells were treated with vehicle (DMSO) or CFZ (30, 60, or 120 nM) for 48 h. For the cell cycle analysis, the collected cells were fixed in ice-cold 70% (v/v) ethanol overnight, followed by staining with propidium iodide (PI; BD Biosciences, San Jose, USA) for 30 min in the dark. For the apoptosis assay, cells were collected and stained with Annexin V-FITC/PI for 20 min at room temperature. An Accuri ^TM^ C6 plus flow cytometer system (BD Biosciences) was used to analyze the cell-cycle distribution and apoptosis.

### Intracellular and mitochondrial ROS assay

The levels of intracellular and mitochondrial ROS were examined using the fluorescent probe 2′,7′-dichlorodihydrofluorescein diacetate (DCFH-DA; Beyotime Biotechnology) and MitoSOX ^TM^ Red (Thermo Fisher Scientific) according to the manufacturer’s instructions. The results were obtained by the Accuri ^TM^ C6 plus flow cytometer system.

### Western blot analysis

Cells or tumor tissues were lysed in lysis buffer (50 mM Tris-HCl, pH 7.4, 1% Triton X-100, 0.1% SDS, and 150 mM NaCl) containing protease inhibitor cocktail (APExBIO) and phosphatase inhibitors (1 mM NaF and 1 mM Na _3_VO _4_) for 20 min on ice, and then centrifuged at 12,000 *g* for 20 min at 4°C. Protein concentrations were determined using the BCA protein assay kit (Pierce, Rockford, USA). Total protein samples were separated by 10% SDS-PAGE and transferred to nitrocellulose membranes (Amersham Biosciences, Piscataway, USA). The membranes were blocked in 5% nonfat milk (BD Bioscience) at room temperature for 1 h and then cut horizontally, and incubated with primary antibodies at 4°C overnight. The next day, the membranes were washed three times (5 min each) with Tris buffered saline supplemented with Tween 20 (TBST), and subsequently incubated with an HRP-conjugated secondary antibody (ProteinTech) for 1 h at room temperature. The membranes were then washed three times (10 min each) and incubated with ECL solution (Thermo Fisher Scientific). The protein bands were visualized by exposure to X-ray film and quantified by ImageJ Plus.

### RNA isolation and quantitative real-time PCR (qPCR)

RNA isolation and qPCR analysis were performed as previously described [17]. Briefly, the total RNA (1 μg) of each sample was used for reverse transcription using HiScript II Q RT SuperMix (Vazyme, Nanjing, China) according to the manufacturer’s instructions. qPCR assay was carried out with the CFX ConnectTM real-time system (Bio-Rad) using the SYBR Green kit (Bio-Rad). The following program was used: 95°C for 10 min, followed by 40 cycles of denaturation at 95°C for 15 s and extension at 60°C for 30 s. The 2 ^‒ΔΔCt^ method was used to quantify the relative levels of mRNA, with *β-Actin* serving as the house-keeping gene. Τhe primer sequences for qPCR are shown in Table 1.

**Table:** **Table 1** Sequences of primers used in qPCR analysis

Gene	Primer sequence (5′→3′)
*ATF4*	Forward: CCAACAACAGCAAGGAGGAT
	Reverse: AGGTCATCTGGCATGGTTTC
*BiP*	Forward: CATCACGCCGTCCTATGTCG
	Reverse: CGTCAAAGACCGTGTTCTCG
*CHOP*	Forward: GGAAACAGAGTGGTCATTCCC
	Reverse: CTGCTTGAGCCGTTCATTCTC
*IRE1α*	Forward: CCTCTATGCCTCTCCCTCAA
	Reverse: ATCACACACTCCCCCTTGTC
*β-Actin*	Forward: AGCACAGAGCCTCGCCTTTG
	Reverse: AAGCCGGCCTTGCACATG

### mtDNA copy number detection

Total DNA was extracted using DNA Extraction Kit (Thermo Fisher Scientific) according to the manufacturer’s instructions, and the mtDNA copy numbers were measured using the SYBR Green kit (Bio-Rad) on the CFX Connect ^TM^ real-time system (Bio-Rad). The primer sequences for mtDNA ( *Cyt b*) and for the internal control (18S ribosomal DNA) are as follows: *Cyt b*-Forward 5′-CCCCACAAACCCCATTACTAAACCCA-3′, *Cyt b*-Reverse 5′-TTTCATCATGCGGAGATGTTGGATGG-3′; *18S ribosomal DNA*-Forward 5′-TAGAGGGACAAGTGGCGTTC-3′, *18S ribosomal DNA*-Reverse 5′-CGCTGAGCCAGTCAGTGT-3′.

### *In vivo* subcutaneous xenograft model

All animal experiments were conducted in accordance with ARRIVE guidelines for the Care and Use of Laboratory Animals on a protocol approved by the Institutional Animal Care and Use Committee, University Laboratory Animal Research of Wenzhou Medical University. Male BALB/c nude mice (4-week-old; SLAC Laboratory Animal, Shanghai, China) were housed under specific pathogen-free conditions in the experimental animal facility. A total of 5×10 ^6^ HCCLM3 cells were suspended in 100 μL PBS, and then injected subcutaneously into the left flank of the nude mice ( *n*=8). When the average tumor volume reached approximately 100 mm ^3^, the mice were randomly divided into two groups. The tumor-bearing mice were intraperitoneally injected with 0.9% NaCl alone or CFZ (4 mg/kg) dissolved in 0.9% NaCl, once a day for 12 days. The body weight and tumor size were monitored every other day to measure the growth of the tumor. At the indicated time points, the tumor volume was evaluated according to the formula 1/2× *L*× *W* ^2^, with *L* denoting the longest superficial diameter and *W* the shortest [19]. At the end of treatment on day 12, all of the nude mice were sacrificed and the tumors were dissected, weighed, photographed, and subject to immunohistochemistry analysis.

### Immunohistochemistry (IHC) staining

Paraffin-embedded tissue sections were deparaffinized twice in xylene, and rehydrated with an alcohol gradient, followed by incubation with 3% hydrogen peroxide for 15 min at room temperature. Then, nonspecific binding was blocked with 5% BSA in PBS for 45 min. Subsequently, the tissue sections were incubated overnight at 4°C with anti-Ki67 and anti-PCNA antibodies. Then, the sections were further washed three times (10 min each) with TBST, followed by incubation with biotin-labeled goat anti-rabbit IgG secondary antibody (SABC POD; Boster, Wuhan, China). After further incubation with streptavidin-HRP (Boster) for 45 min at room temperature, 3,3′-diaminobenzidine was used to visualize the signals of the IHC staining, and the sections were lightly counterstained with Mayer’s hematoxylin solution (Solarbio). The positively labeled cells (brown) were quantified by densitometric analysis using ImageJ Plus.

### Statistical analysis

All experiments were performed in triplicate and repeated independently at least three times. All statistical analyses were carried out using SPSS software version 22.0. Student’s *t* test was used for two group comparisons, and one-way ANOVA followed by Tukey’s *post hoc* test was used for multiple-group comparisons of one independent factor. The graphs were created by GraphPad Prism 7.0 Plus software. All data were presented as the mean±SD. *P*<0.05 was considered statistically significant.

## Results

### CFZ markedly suppresses HCC cell growth *in vitro*

**Figure 1:**
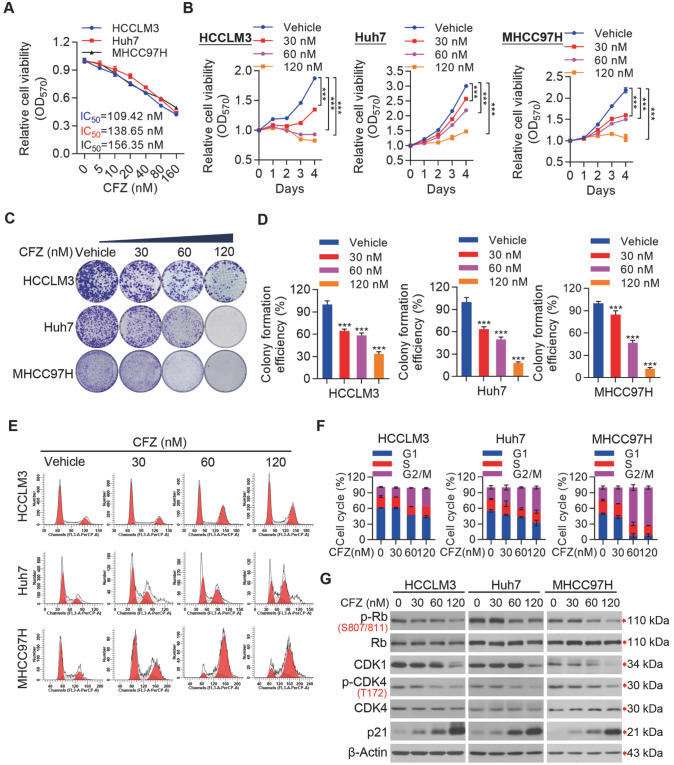
Figure 1 CFZ suppresses HCC cell growth *in vitro* (A) Cell viability of HCC cells and the IC50 was measured by the CCK-8 assay. (B) CCK-8 assay was used to measure the proliferation of HCC cells treated with the indicated concentrations of vehicle or CFZ. (C,D) Representative images (C) and quantitative data (D) of colony formation assays of HCC cells treated with or without CFZ. (E,F) Cell-cycle distributions were analyzed by flow cytometry (E) and CFZ-induced cell cycle arrest at the G2/M phase in HCC cells, compared with vehicle treatment (F). (G) Western blot analysis for cell cycle-related proteins in HCC cells treated with indicated concentrations of vehicle or CFZ. Data are presented as the mean±SD (n=3). Group comparisons were performed by Student’s t test (B) and one-way ANOVA followed by Tukey’s post hoc test (D). ***P<0.001.

To evaluate the specific effect of CFZ on HCC cell growth, we performed cell viability and proliferation assays. We found that CFZ markedly inhibited HCC cell viability and proliferation in a dose-dependent manner ( Figure 1A,B). At the same time, we conducted a colony formation assay to explore the antiproliferative activity of CFZ on HCC cells. The results showed that CFZ significantly reduced the clonogenic ability of HCC cells ( Figure 1C,D). Additionally, CFZ markedly arrested HCC cells at G2/M phase ( Figure 1E,F). To further understand the mechanism of CFZ in suppressing HCC cell proliferation, we detected the expression of cell cycle-related proteins in HCC cells after treatment with CFZ. We found that CDK1 and p-CDK4/CDK4 were significantly reduced in HCC cells ( Figure 1G). Moreover, p-Rb/Rb was remarkably reduced, while p21 protein expression was significantly increased ( Figure 1G). Together with the aforementioned results, our data demonstrated that CFZ suppresses HCC cell growth *in vitro* at least partially by arresting the cell cycle.

### CFZ dramatically suppresses HCC cell growth in a mouse xenograft model

**Figure 2:**
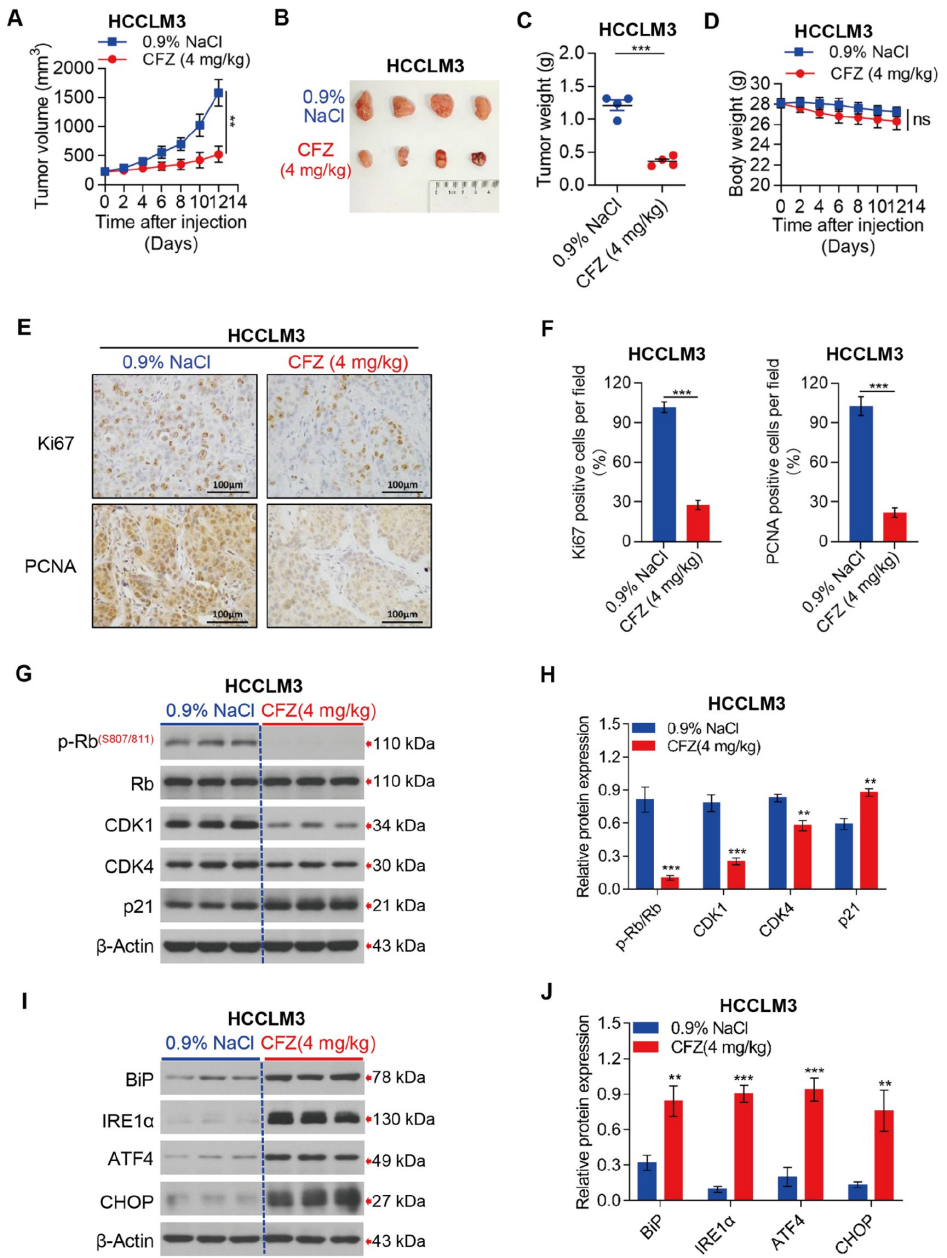
Figure 2 CFZ suppresses HCC tumor growth *in vivo* (A–D) HCCLM3 cells were used for establishing a xenografted nude mice model. Nude mice bearing HCCLM3 cell tumors (n=4) were injected with either CFZ (4 mg/kg) or 0.9% NaCl. Tumor volumes were monitored at the indicated time points (A). Data are presented as the mean±SD (n=4). The mice were sacrificed to harvest tumors after 12 days of once-daily injections. Representative images of dissected tumors are shown (B) and the tumors were weighed (C). Data are presented as the mean±SD (n=4). To assess the effect of CFZ on body weight, we measured the body weight of mice treated with CFZ (4 mg/kg) or 0.9% NaCl at the indicated time intervals (D). Data are presented as the mean±SD (n=4). (E,F) The expressions of cell proliferation markers Ki67 and PCNA in tumors from xenografted nude mice treated with CFZ (4 mg/kg) or 0.9% NaCl was determined by IHC staining (E) and Ki67- and PCNA-positive staining cells were quantified using ImageJ Plus (F). (G,H) The expressions of cell cycle-related proteins were detected by western blot analysis (G), and p-Rb/Rb, CDK1, CDK4 and p21 levels were quantified using ImageJ Plus (H). (I,J) Western blot analysis was used to examine the expressions of UPRER-related proteins (I), and BiP, IRE1α, ATF4 and CHOP levels were quantified using ImageJ Plus (J). Data are presented as the mean±SD (n=3). Student’s t test was used to determine the differences. **P<0.01, ***P<0.001. ns, not significant.

Next, we investigated the antitumor activity of CFZ *in vivo* using a mouse xenograft model. Consistent with the findings *in vitro*, CFZ remarkably inhibited HCC tumor growth compared to the control *in vivo* ( Figure 2A,B). In line with these results, we observed that CFZ significantly reduced the tumor weight compared to the control ( Figure 2C). Of note, we also observed that the mice were well tolerated to CFZ treatment, and there was no notable body weight loss throughout CFZ treatment, suggesting that CFZ had few side effects ( Figure 2D). Furthermore, IHC staining showed that CFZ treatment reduced the expression of proliferation markers (Ki67 and PCNA) in HCC xenograft tumor sections ( Figure 2E,F). We further analyzed cell cycle-related proteins in tumor tissues from saline- and CFZ-treated groups. In agreement with previous studies, CFZ treatment resulted in a dramatic downregulation in the expressions of CDK1, p-CDK4/CDK4 and p-Rb/Rb, with a concomitant upregulation in the expression of p21 ( Figure 2G,H). Moreover, we found that CFZ treatment led to the upregulation of BiP, IRE1α, ATF4 and CHOP protein expressions ( Figure 2I,J). Collectively, these findings suggested that CFZ significantly suppresses HCC cell growth *in vivo*.

### CFZ suppresses the migration and invasion of HCC cells *in vitro*

**Figure 3:**
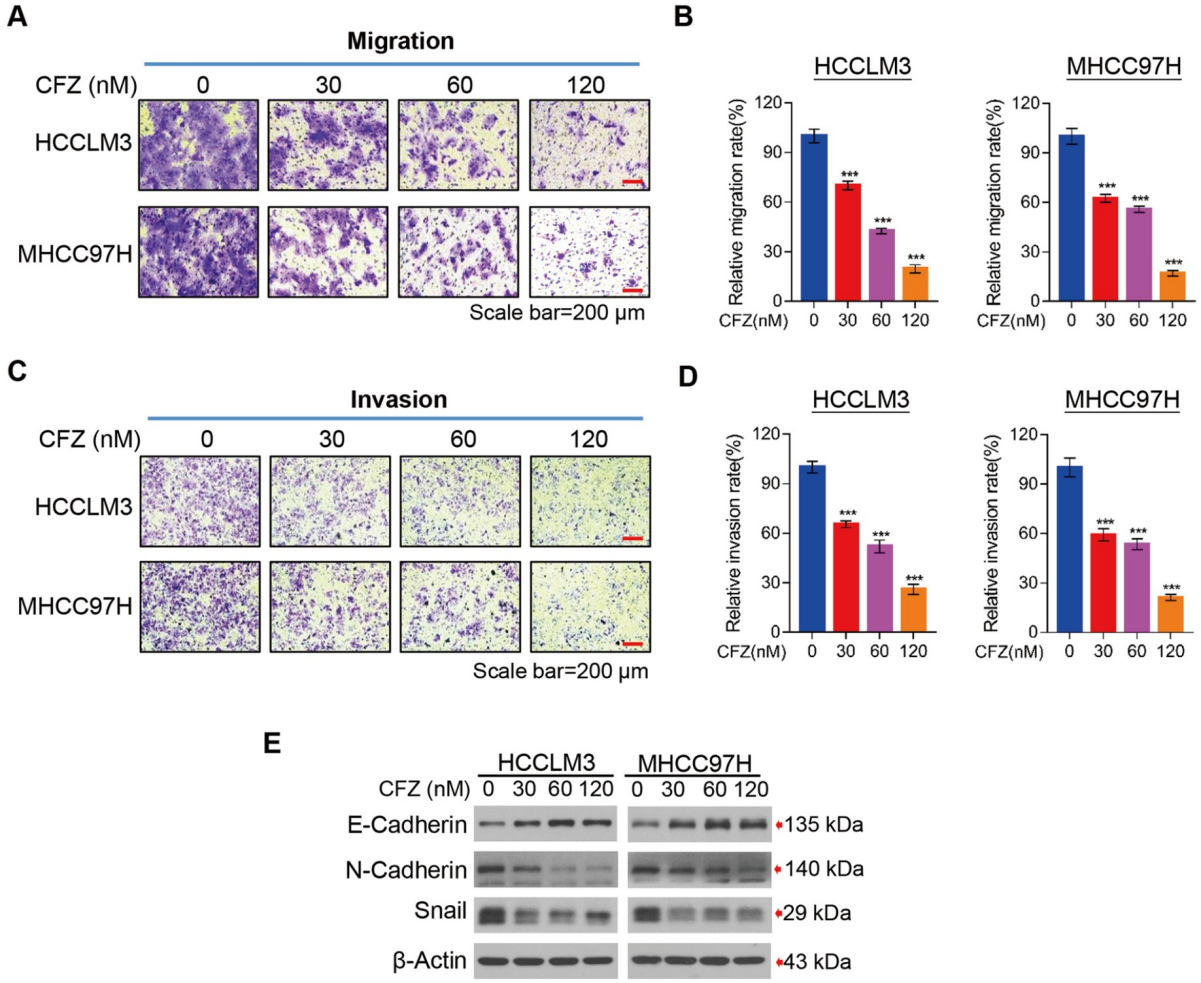
Figure 3 CFZ suppresses HCC cell migration and invasion (A‒D) Transwell assays for the effect of CFZ on HCC cell migration and invasion. Representative images (A,C) and quantitative analysis of cell migration and invasion (B,D) of HCCLM3 and MHCC97H cells. (E) HCCLM3 and MHCC97H cells were treated with CFZ or DMSO for 48 h, and the cell migration- and invasion-related proteins were analyzed by western blot analysis. Data are presented as the mean±SD (n=3). ***P<0.001 by one-way ANOVA followed by Tukey’s post hoc test.

Metastasis is closely associated with high mortality of HCC patients. Next, we investigated the effectiveness of CFZ treatment to inhibit the migration and invasion of HCC cells. First, we observed that CFZ dramatically suppressed HCC cell migration in a dose-dependent manner *in vitro* ( Figure 3A,B). Then, we examined the impact of CFZ on HCC cell invasion. In agreement with the migration data, CFZ also significantly inhibited HCC cell invasion in a dose-dependent manner *in vitro* ( Figure 3C,D). To further explore the underlying mechanisms on how CFZ inhibits the migration and invasion abilities of HCC cells, EMT-related protein expression was tested in HCC cells. The data clearly showed that CFZ remarkably reduced the expression of migration and invasion-related markers including N-cadherin and Snail in HCC cells. In contrast to N-cadherin, E-cadherin, an anti-metastatic protein, was upregulated in a dose-dependent manner ( Figure 3E). Altogether, these findings indicate that CFZ inhibits the migration and invasion of HCC cells through regulating the EMT process.

### CFZ impairs mitochondrial function and induces ROS production in HCC cells

**Figure 4:**
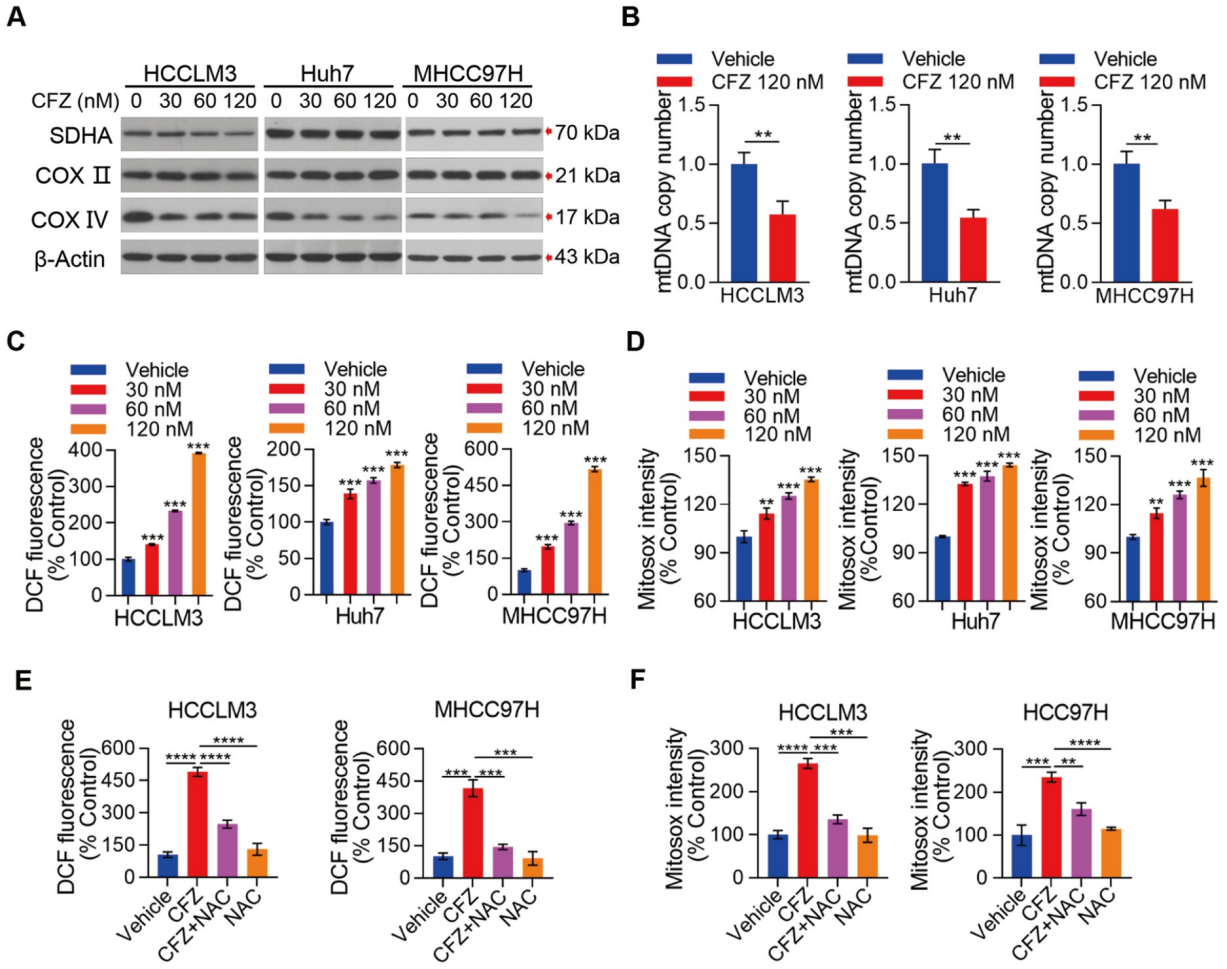
Figure 4 CFZ impairs mitochondrial function and increases ROS generation in HCC cells (A) HCC cells were incubated with vehicle or CFZ for 48 h, and the indicated proteins were assessed by western blot analysis. (B) mtDNA copy numbers were measured in HCC cells treated with CFZ (120 nM) or vehicle for 48 h. (C,D) DCFH-DA staining of total ROS (C) and MitoSOX Red staining of mitochondrial ROS (D) were examined by flow cytometry in HCC cells treated with vehicle or CFZ for 48 h. (E,F) DCFH-DA staining of total ROS (E) and MitoSOX Red staining of mitochondrial ROS (F) were examined by flow cytometry in HCC cells treated with DMSO, CFZ, CFZ combined with NAC, or NAC alone for 48 h. Data are expressed as the mean SD (n=3). Group comparisons were performed by Student’s t test (B, E, and F) and one-way ANOVA followed by Tukey’s post hoc test (C,D). **P<0.01, ***P<0.001, ****P<0.0001.

It is generally known that cellular energy (ATP) metabolism is critical for cell growth, and that ATP is mainly produced through mitochondrial oxidative phosphorylation (OXPHOS). Growing evidence suggests that targeting OXPHOS is a potential therapeutic approach for the treatment of cancer. In this work, we demonstrated that CFZ treatment decreased the protein level of COX IV which is one of the subunits comprising the complex IV of mitochondrial respiratory chain in a dose-dependent manner. However, the expressions of COX II (a subunit of complex IV) and SDHA (a subunit of complex II) remained unchanged ( Figure 4A). Furthermore, accumulating evidence suggests that the proportion of mutant mtDNA is not the only determinant in various types of mitochondrial diseases, the mtDNA copy number is also a determinant factor [20]. In this study, we found that CFZ treatment reduced mtDNA copy number in HCC cells ( Figure 4B). Given that the production of ROS is mainly due to mitochondrial dysfunction in eukaryotic cells, we speculated that the mitochondrial dysfunction induced by CFZ would further promote ROS generation. We subsequently analyzed the intracellular and mitochondrial ROS, and in line with the hypothesis, our results showed that CFZ treatment significantly enhanced the generation of both intracellular and mitochondrial ROS in HCC cells ( Figure 4C,D). Pretreatment with NAC remarkably attenuated the effect of CFZ on promotion of both cellular and mitochondrial ROS production in HCC cells ( Figure 4E,F). Taken together, these data indicate that mitochondrial respiration damage promotes ROS production, which leads to the inhibition of HCC cell growth after CFZ treatment.

### CFZ activates IRE1α-dependent ER stress and JNK/p38 MAPK signaling to promote apoptosis in HCC cells

**Figure 5:**
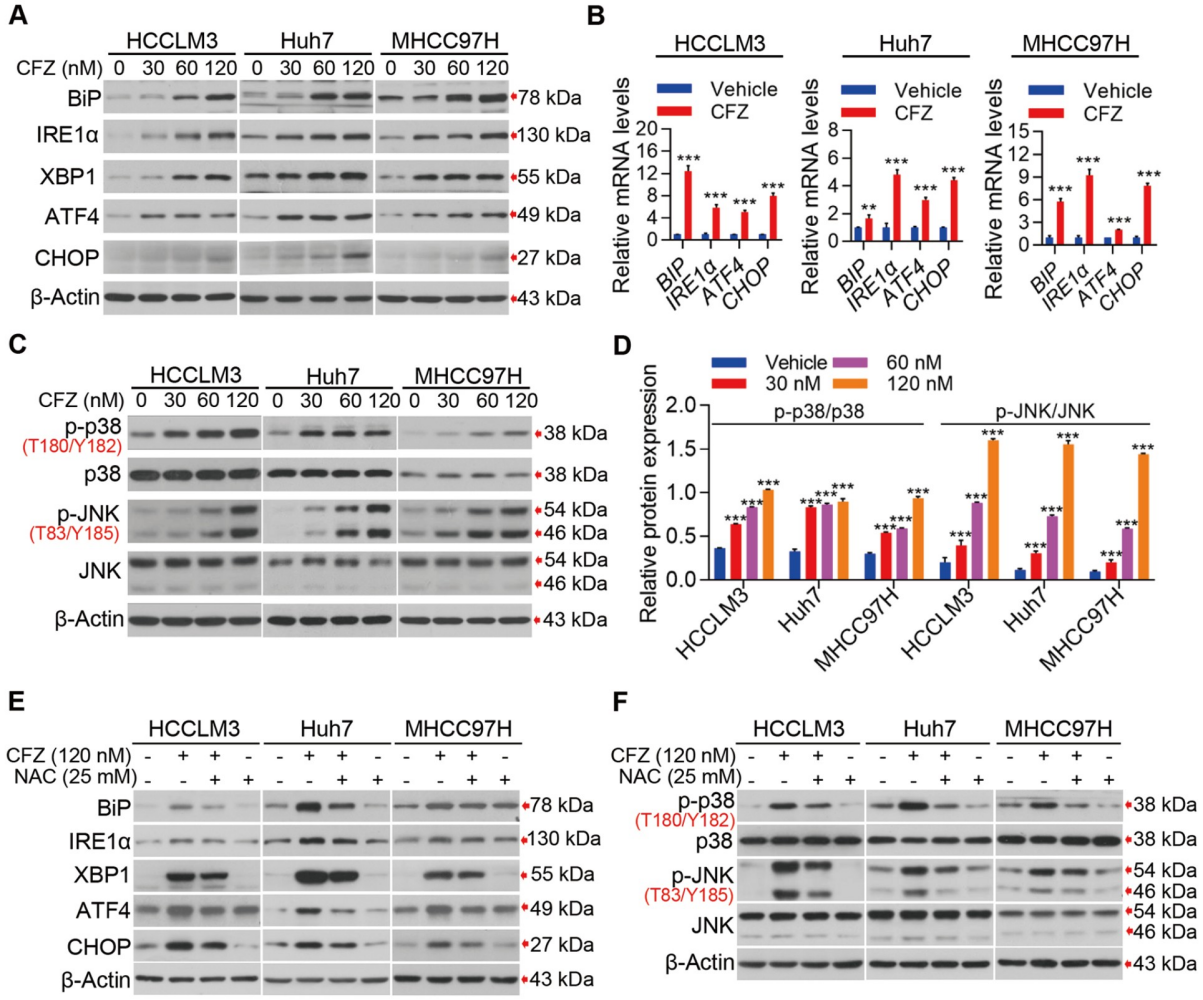
Figure 5 CFZ induces ROS to activate IRE1α-mediated ER stress and JNK/p38 MAPK signaling (A) The IRE1α-mediated ER stress-related proteins in HCC cells treated with vehicle or different concentrations of CFZ were assessed by western blot analysis. (B) The mRNA levels of the BiP, IRE1α, ATF4 and CHOP in vehicle or CFZ (120 nM)-treated HCC cells. (C,D) HCCLM3, Huh7 and MHCC97H cells were treated with vehicle or CFZ for 48 h, followed by western blot analysis to detect the protein expression of JNK/p38 MAPK signaling pathway (C) and the relative expressions of p-p38/p38 and p-JNK/JNK were quantified (D). (E,F) HCCLM3, Huh7 and MHCC97H cells with the treatment of vehicle, CFZ (120 nM) combined with NAC (25 mM) or NAC (25 mM) alone for 48 h. Western blot analysis was performed to detect the expression levels of ER stress-related proteins (E) and proteins of JNK/p38 MAPK signaling pathway (F). Data are expressed as the mean±SD (n=3). Group comparisons were performed by Student’s t test (B) and one-way ANOVA followed by Tukey’s post hoc test (D). **P<0.01, ***P<0.001.

To gain insights into the potential mechanism by which CFZ inhibits HCC cell growth, we further investigated the effects of CFZ on ER stress in HCC cells. Strikingly, the results suggested that CFZ activated IRE1α-dependent ER stress in HCC cells, and the protein expressions of IRE1α, BiP, XBP1, ATF4 and CHOP are significantly increased in a dose-dependent manner ( Figure 5A). Consistently, CFZ treatment also increased the mRNA levels of *BiP*, *IRE1α*, *ATF4* and *CHOP* ( Figure 5B). Moreover, we found that CFZ treatment significantly upregulated the protein levels of p-p38 and p-JNK ( Figure 5C,D). Additionally, treatment of HCC cells with NAC, an ROS scavenging agent, rescued the effects of CFZ on the protein expressions of ATF4, BiP, IRE1α, XBP1 and CHOP ( Figure 5E), as well as the phosphorylation of p38 and JNK ( Figure 5F).

**Figure 6:**
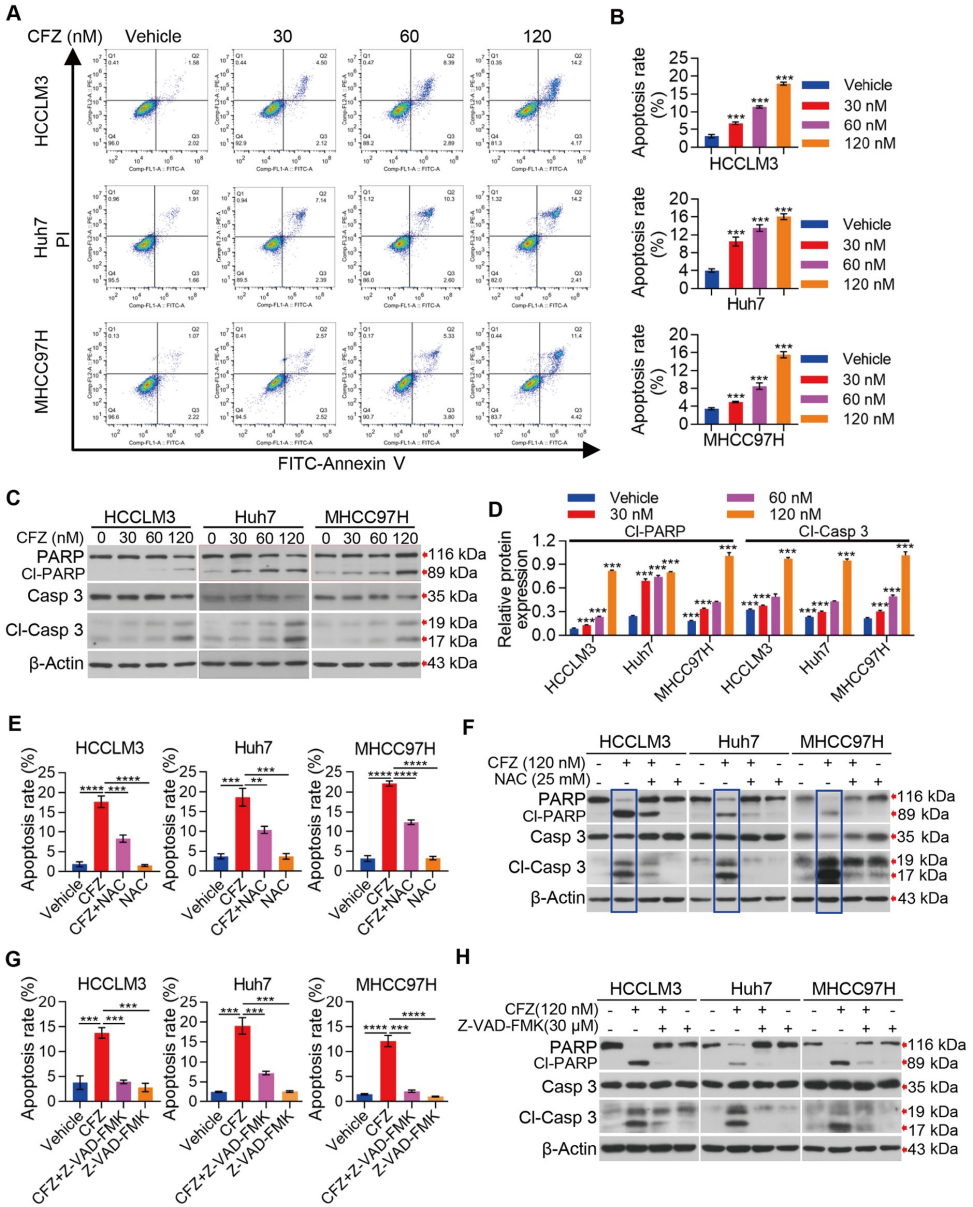
Figure 6 CFZ promotes ROS-dependent apoptosis in HCC cells (A) HCC cells were treated with vehicle or indicated concentrations of CFZ for 48 h, and the apoptosis was measured by flow cytometry. (B) Quantitation of the apoptosis rate is shown. (C,D) HCC cells were treated with the indicated concentration of CFZ for 48 h, followed by western blot analysis of the expression levels of apoptosis-related proteins (C) and the relative expressions of Cl-PARP and Cl-Casp 3 were quantified (D). (E,F) HCC cells with the treatment of vehicle, CFZ (120 nM), CFZ (120 nM) combined with NAC (25 mM) or NAC (25 mM) alone for 48 h. The apoptosis of HCC cells was measured by flow cytometry (E) and the proteins related to apoptosis were detected by western blot analysis (F). (G,H) HCC cells with the treatment of vehicle, CFZ (120 nM), CFZ (120 nM) combined with Z-VAD-FMK (30 μM) or Z-VAD-FMK (30 μM) alone for 48 h. The apoptosis of HCC cells was measured by flow cytometry (G) and the proteins related to apoptosis were detected by western blot analysis (H). Data are expressed as the mean±SD (n=3). One-way ANOVA followed by Tukey’s post hoc test analysis was used for statistical comparison between different groups (B,D) and two-group comparisons were performed by Student’s t test (E,G). **P<0.01, ***P<0.001, ****P<0.0001.

Previous studies reported that the ROS-mediated ER stress and JNK/p38 MAPK signaling are important mechanisms for inducing cell apoptosis [ 21– 23]. Next, we investigated the effects of CFZ on the apoptosis of HCC cells by flow cytometry. Our data showed that CFZ treatment induced the apoptosis of HCC cells in a dose-dependent manner ( Figure 6A,B). We next determined the protein levels of cleaved-PARP and cleaved-caspase-3, which are critical for apoptosis. Our data showed that CFZ treatment increased the cleavage of caspase-3 and PARP in a dose-dependent manner ( Figure 6C,D), and these findings were consistent with the results of flow cytometry ( Figure 6A,B). Moreover, the treatment of NAC also rescued CFZ-induced apoptosis in HCC cells ( Supplementary Figure S1A) as well as the cleavage of caspase-3 and PARP ( Figure 6E,F). Moreover, treatment of HCC cells with Z-VAD-FMK, a cell-permeable and irreversible pan-caspase inhibitor, also suppressed the CFZ-induced apoptosis ( Supplementary Figure S1B) as well as the activation of caspase-3 and PARP ( Figure 6G,H), which further demonstrated that CFZ induced apoptosis in HCC cells. Altogether, these results imply that CFZ inhibits HCC cell growth, at least in part, via inducing ER stress and activating JNK/p38 MAPK signaling to induce apoptosis.

## Discussion

Here, we demonstrated that CFZ significantly inhibited the proliferation and colony formation ability of HCC cells. Mechanistically, we found that CFZ reduced the CDK1 and p-CDK4 protein levels, which led to cell cycle arrest at G2/M phase. Meanwhile, CFZ inhibited the phosphorylation of Rb, and increased p21 expression in HCC cells to inhibit cell proliferation. Our findings provided strong evidence that CFZ could suppress HCC cell growth *in vitro*. We further demonstrated the antitumor ability of CFZ in a mouse xenograft tumor model of HCC, as our data indicated that CFZ treatment markedly inhibited tumor growth *in vivo*. Moreover, it was confirmed that Ki67 and PCNA act as proliferation markers in several malignant tumors [24]. Correspondently, our results showed that CFZ treatment remarkably reduced Ki67 and PCNA expressions in tumor tissues of mouse xenograft model of HCC, compared with the control treatment.

The set of events converting adherent epithelial cells into migratory cells are known as EMT, which is a transformation process that is mandatory for the local and distant progression of many malignant tumors, including HCC [ 25, 26]. The hallmark of EMT is the upregulation of N-cadherin followed by the downregulation of E-cadherin, and this process is regulated by a complex network of signaling pathways and transcription factors, including Snail, which is a zinc-finger transcriptional repressor controlling EMT during embryogenesis and tumor progression [ 27‒ 29]. Our data suggested that CFZ increased E-cadherin protein expression; on the contrary, the expressions of Snail and N-cadherin dramatically decreased in a CFZ dose-dependent manner. This indicated that HCC cell migration and invasion abilities were suppressed through the inhibition of EMT by CFZ. Consistent with these observations, transwell assays revealed that CFZ significantly inhibited the migration and invasion abilities of HCC cells.

OXPHOS is crucial for ATP synthesis, which is a key regulator in the tumor microenvironment, and OXPHOS deficiency plays a significant role in tumorigenesis [ 30, 31]. A variety of anomalies in mitochondrial structure and function reduce ATP production through impairing OXPHOS in cancer cells [32]. In this study, we found that CFZ significantly downregulated the expression of COX IV, one subunit of complex IV. Complex IV acts as the final and rate limiting step of the mitochondrial respiratory chain, representing the regulatory center of OXPHOS [33]. mtDNA is important for ATP production, as it encodes some of the key proteins of the electron transfer chain, where the majority of ATP is generated through OXPHOS [ 34, 35]. Therefore, we measured the mtDNA copy numbers in CFZ-treated HCC cells, and in agreement with our hypothesis, CFZ treatment dramatically decreased mtDNA copy numbers in HCC cells. Altogether, our data suggest that CFZ inhibits mtDNA replication and gene transcription, further impairing complex IV, thus leading to OXPHOS dysfunction. One consequence of OXPHOS dysfunction is the production of ROS, which causes cellular damage [ 36, 37]. Indeed, our data suggested that CFZ treatment significantly increased cellular and mitochondrial ROS in HCC cells, indicating that CFZ may disrupt the cellular energy metabolism of HCC cells by inducing ROS generation and reducing mitochondrial respiratory chain enzyme complex activity. However, further studies are needed to understand the specific mechanism involved.

The ER is a dynamic organelle that participates in a number of cellular functions. ER stress triggers a series of signaling and transcriptional events, *i.e*. the UPR ^ER^. The UPR ^ER^ protects cells from stress and contributes to cellular homeostasis maintenance; however, during prolonged ER stress, UPR ^ER^ activation promotes cell apoptosis [ 38‒ 40]. Indeed, an increasing number of studies have shown the role of UPR signaling, which is driven namely by IRE1, PERK, and ATF6, in different aspects of carcinogenesis and tumor progression. Thus, targeting ER stress or the UPR ^ER^ is considered an effective therapy for cancers [ 41, 42].

The proteasome plays a critical role in clearing unfolded/misfolded proteins, and previous studies have indicated that bortezomib induces ER stress–mediated apoptosis in multiple myeloma [ 43, 44]. However, the mechanisms underlying the anticancer effect of the proteasome are still not fully understood. In this study, we report that CFZ directly activates IRE1α-dependent ER stress to promote apoptosis in HCC cells. Specifically, CFZ leads to the activation of BiP, a primary sensor in the activation of the UPR. We further investigated the IRE1α-XBP1 axis, one of the three UPR branches, in HCC cells treated with CFZ. We found that CFZ significantly increased the expressions of IRE1α, XBP1 and CHOP. Importantly, caspase-dependent apoptosis was activated simultaneously, as shown by the increase in cleaved-PARP and cleaved-caspase 3.

Mitogen-activated protein kinases (MAPKs) are highly conserved eukaryotic signaling modules, including the extracellular signal-regulated kinase (ERK), p38, and c-Jun NH _2_-terminal kinase (JNK) MAPK subfamilies. MAPKs coordinately regulate a wide range of cellular processes, including cell proliferation, differentiation, metabolism, survival, and apoptosis [ 45‒ 48]. In response to cytokines and various types of extracellular and intracellular stress, the JNK/p38 MAPK signaling pathway is activated to regulate stress-induced cellular responses and a sustained activation of JNK and p38 can contribute to apoptosis [ 49‒ 51]. Therefore, the JNK/p38 MAPKs are also termed stress-activated MAPKs. Previous results indicated that CFZ increases both cellular and mitochondrial ROS in HCC cells. The ROS-mediated ER stress is also related to JNK/p38 MAPK pathway activation. More specifically, ROS accumulation results in the activation of the JNK/p38 MAPK signaling pathway and therefore induces apoptosis [ 23, 52]. In this study, we found that CFZ dramatically increased the levels of phosphorylated p38 MAPK and phosphorylated JNK in a dose-dependent manner. Taken together, our findings revealed a critical role for ROS-mediated ER stress and JNK/p38 MAPK signaling in CFZ-induced apoptosis in HCC cells. However, further investigation is required to validate this hypothesis and to gain further insight into the molecular mechanism by which CFZ activates ER stress and JNK/p38 MAPK signaling to promote apoptosis in HCC cells.

**Figure 7:**
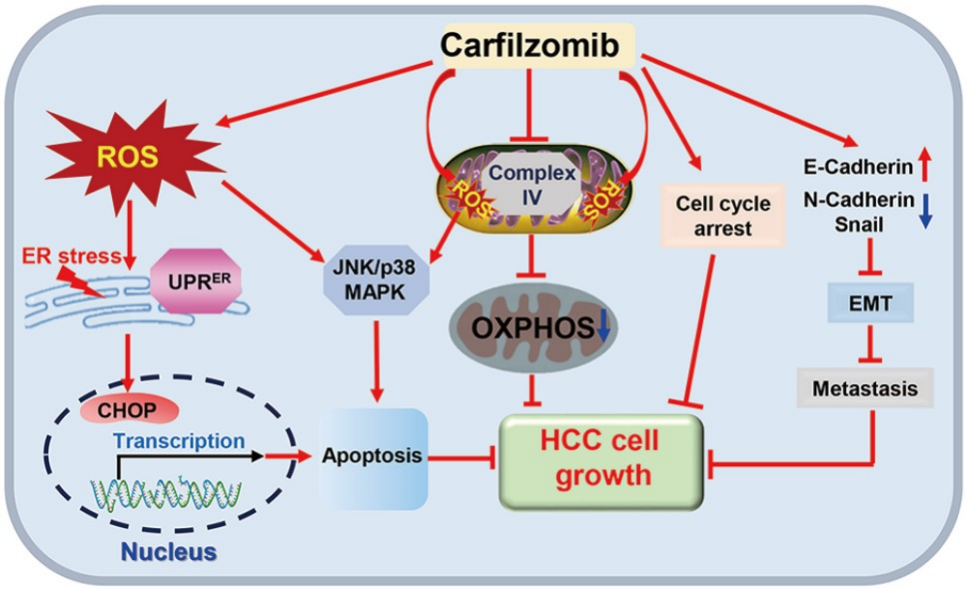
Figure 7 The proposed mechanistic model of CFZ suppressing HCC cell growth

In conclusion, a novel model in which CFZ inhibits HCC cell growth was proposed in this study. Mechanistically, CFZ activates ROS-mediated ER stress and JNK/p38 MAPK signaling to induce apoptosis in HCC cells ( Figure 7). Our findings reveal a novel mechanism by which CFZ suppresses HCC cell growth *in vitro* and *in vivo*. Together with the aforementioned results, our data demonstrate that CFZ is a potential and valuable therapeutic agent for HCC patients.

## Supporting information

553Supplementary_Material

## Supplementary Data

Supplementary data is available at *Acta Biochimica et Biophysica Sinica* online.
