# Supplementary material for: Carfilzomib activates ER stress and JNK/p38 MAPK signaling to promote apoptosis in hepatocellular carcinoma cells: Carfilzomib promotes HCC cell death
Source: Acta Biochim Biophys Sin (Shanghai). 2024 Apr 9;56(5):697–708. doi: 10.3724/abbs.2024040 (PMC11177107; doi:10.3724/abbs.2024040)
Supplement: 553Supplementary_Material [file 553Supplementary_Material.pdf]

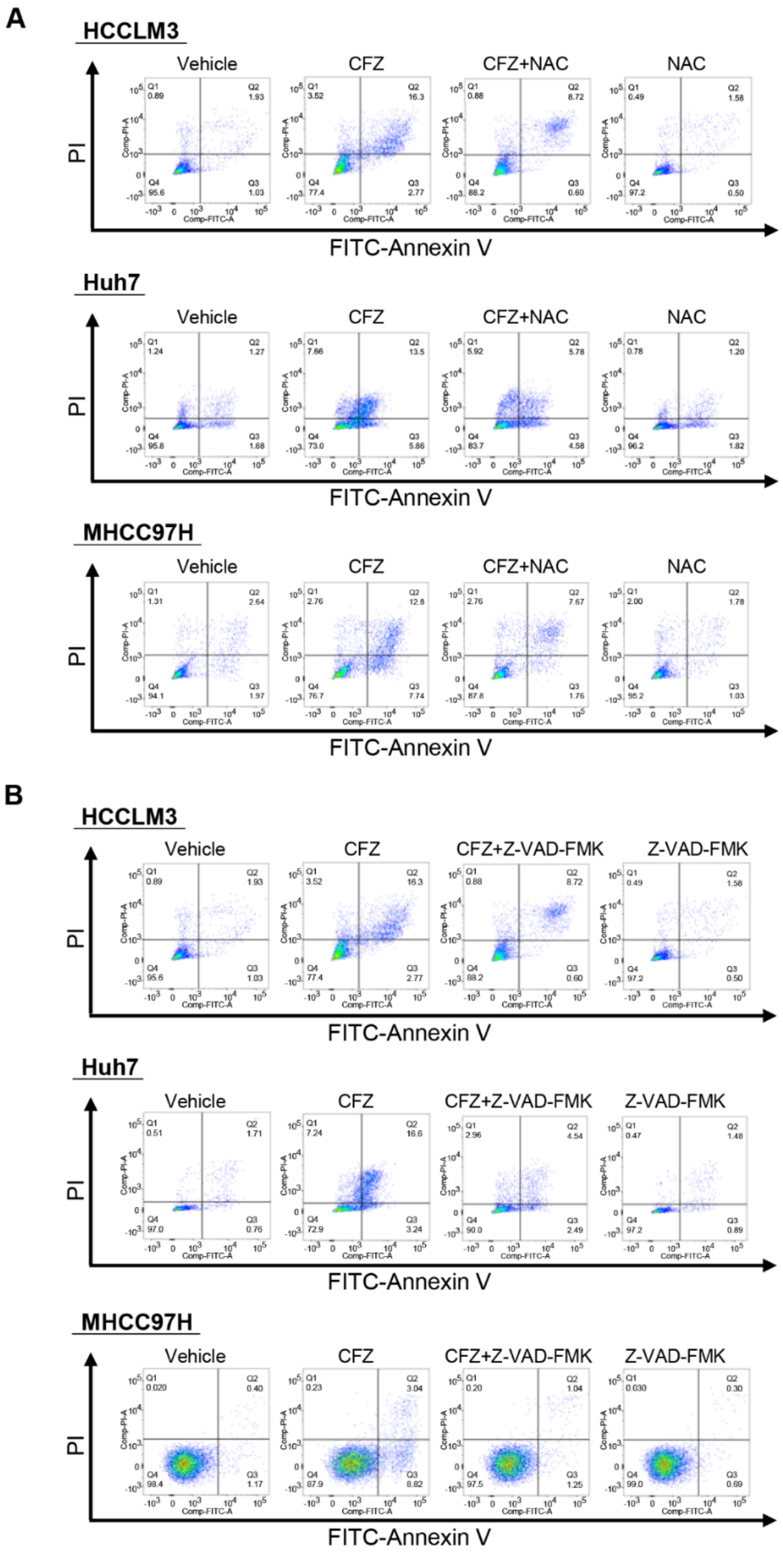

Supplementary Figure S1. CFZ promotes ROS-dependent apoptosis in HCC cell (A) HCC cells were treated with vehicle (DMSO), CFZ (120 nM), the combination of CFZ (120 nM) and NAC (25 mM), or NAC (25 mM) for 48 h, and the apoptosis was measured by flow cytometry. (B) HCC cells were treated with vehicle (DMSO), CFZ (120 nM), the combination of CFZ (120 nM) and Z-VAD-FMK (30 μM), or Z-VAD-FMK (30 μM) for 48 h, and the apoptosis was measured by flow cytometry.
